# Circulating neuregulin 4 levels are inversely associated with subclinical cardiovascular disease in obese adults

**DOI:** 10.1038/srep36710

**Published:** 2016-11-07

**Authors:** Jie Jiang, Mingzhu Lin, Yanfang Xu, Jin Shao, Xuejun Li, Huijie Zhang, Shuyu Yang

**Affiliations:** 1 Xiamen Cancer Center, Department of Thoracic Surgery, The First Affiliated Hospital of Xiamen University, Xiamen, China; 2 Department of Endocrinology and Diabetes, The First Affiliated Hospital of Xiamen University, Xiamen, China; 3 Department of Nephrology, The First Affiliated Hospital, Fujian Medical University, Fuzhou, China; 4 Department of Epidemiology, Tulane University Health Sciences Center, New Orleans, LA

## Abstract

Neuregulin 4 (Nrg4) has been identified as a new secreted adipokine that may protect against development of obesity and metabolic disorders. However, information is not available regarding the association between circulating Nrg4 and subclinical atherosclerosis in humans. We measured serum Nrg4 in 485 obese adult subjects (aged 40 years or older) who had the measurement of carotid intima-media thickness (CIMT) recruited from the community. Individuals with increased CIMT and carotid plaque had lower levels of circulating Nrg4 than controls (p < 0.05). The risks of increased CIMT and atherosclerotic plaque were significantly decreased by 28% and 31% [OR (95% CI): 0.72 (0.53–0.98) and 0.69 (0.50–0.96), respectively], adjusting for age, sex, current smoking, alcohol consumption, physical activity, BMI, systolic BP, fasting glucose, total cholesterol, HDL-c, HOMA-IR, and body fat. Importantly, individuals in the lowest quartile of serum Nrg4 were 3.70 times (p < 0.001) more likely to have increased CIMT and 2.06 times (p < 0.05) more likely to have atherosclerotic plaque than those in the highest quartile in multivariable logistic regression analyses. These findings suggest that circulating Nrg4 concentrations are inversely associated with subclinical atherosclerosis in obese adults, and indicating that circulating Nrg4 might play a role in identifying patients at high risk for CVD.

Cardiovascular disease (CVD) is the leading cause of morbidity and mortality worldwide^1^. It affects approximately 230 million people in China, accounting for 40% of all deaths in 2011^2^,^3^. Identification of risk factors for the development of CVD could aid development of prevention strategies. Numerous risk factors have been well established, including age, smoking, physical inactivity, obesity, hypertension, diabetes, and hyperlipidemia^4^,^5^,^6^. Increasing evidence from epidemiological studies has highlighted the association of excess adiposity with increased risk of CVD^5^,^7^. Insulin resistance is likely to account for some, but not all, of this association^8^.

The mechanisms by which insulin resistance and obesity promote CVD risk remain uncertain. Proposed mechanisms include a systemic inflammatory state with increased concentrations of circulating inflammatory mediators such as C-reactive protein, interleukin-6, tumor necrosis factor-alpha, and vascular endothelial growth factor^9^,^10^,^11^. Several novel proteins secreted by adipocytes (adipokines), including resistin and adiponectin, have pro- and anti-flammatory properties and are associated with risk of CVD^11^,^12^,^13^. Recently, secreted adipokine neuregulin 4 (Nrg4) has been identified as playing an important role in modulating systemic energy metabolism and in the development of obesity-associated disorders, including type 2 diabetes and nonalcoholic fatty liver disease (NAFLD)^14^. As a brown fat-enriched endocrine factor, Nrg4 attenuates hepatic lipogenic signaling and preserves glucose and lipid homeostasis in obesity^15^. Given the connection between obesity-associated metabolic disorder and risk of CVD, circulating Nrg4 might be a candidate marker of CVD risk. However, to date, the association between circulating Nrg4 and risk of CVD has not yet been studied in humans.

Carotid intima-media thickness (CIMT) measured by ultrasound is widely used as a valid noninvasive measurement for subclinical atherosclerotic diseases and predicts CVD events in the general population^16^,^17^. Information is not available regarding the association of circulating Nrg4 with CIMT and atherosclerotic plaque in humans. In the current study, we aimed to explore the association between circulating Nrg4 and subclinical atherosclerosis in obese Chinese adults.

## Results

Table 1 summarizes the mean levels of study variables in obese subjects. The mean age of the subjects was 54.2 ± 7.2 years. Male subjects had significantly higher levels of BMI, waist circumference, systolic BP, diastolic BP, and triglycerides than female subjects. Also, male subjects had significantly lower levels of HDL-c, postprandial glucose, and body fat percent than female subjects. There were no sex differences in total cholesterol, LDL-c, fasting plasma glucose, and HOMA-IR. Furthermore, male subjects had higher prevalence of carotid plaque and higher values of CIMT than female subjects (p < 0.05). Of interest, male subjects had significantly lower serum Nrg4 levels than female subjects [median (interquartile range), 3.2 ng/ml (2.3–4.9) vs.3.9 ng/ml (2.8–6.4), p < 0.05].

Supplemental Figure 1 shows serum Nrg4 levels by quartiles of CIMT. Serum Nrg4 levels gradually decreased across increasing quartiles of CIMT (p for trend < 0.05). Additionally, mean levels of serum Nrg4 by presence of carotid plaque are shown in Supplemental Figure 2. Subjects with carotid plaque had lower levels of serum Nrg4 than healthy controls (p < 0.05).

Table 2 presents clinical characteristic by quartiles of serum Nrg4 levels. There were no differences in age, physical activity, diastolic BP, total cholesterol, LDL-c, HDL-c, fasting glucose, postprandial glucose, body fat percent, and HOMA-IR among four quartiles of serum Nrg4 levels. Compared to subjects in the lowest quartile of serum Nrg4 levels, those in the highest quartile had significantly lower levels of BMI, total cholesterol, and systolic BP (p < 0.05). Lower levels of serum Nrg4 were associated with higher prevalence of carotid plaque (p < 0.05). Furthermore, CIMT gradually decreased from quartile 1 to quartile 4 (0.78 ± 0.17 mm, 0.77 ± 0.14 mm, 0.73 ± 0.15 mm, and 0.66 ± 0.12 mm, respectively).

**Figure 1:**
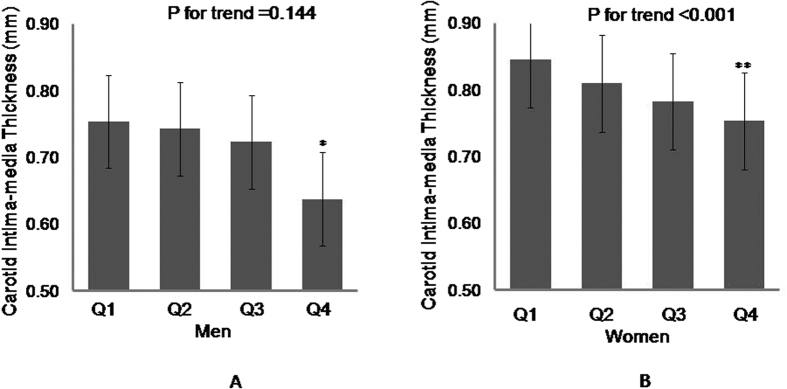
Carotid intima-media thickness according to serum neuregulin 4 levels by sex. (**A**) Carotid intima-media thickness according to serum neuregulin 4 levels among male subjects (**B**) Carotid intima-media thickness according to serum neuregulin 4 levels among female subjects; *P < 0.05 compared with Q1; **P < 0.01 compared with Q1; CIMT = carotid intima-media thickness.

As shown in Fig. 1, CIMT was inversely correlated with serum Nrg4 levels in males and females. In female subjects, CIMT significantly decreased across increasing quartiles of serum Nrg4 levels (0.75 ± 0.15 mm in quartile 1, 0.74 ± 0.14 mm in quartile 2, 0.72 ± 0.16 mm in quartile 3, and 0.64 ± 0.11 mm in quartile 4, p for trend <0.001). In male subjects, CIMT also showed a tendency to decrease when serum Nrg4 levels increased gradually (p = 0.144); male subjects in the highest quartile of serum Nrg4 levels had significantly lower levels of CIMT than those in the lowest quartile (p < 0.05).

Table 3 presents results of linear regression analyses of serum Nrg4 levels and metabolic risk factors and CIMT. In multivariable linear regression models, serum Nrg4 levels, systolic BP, total cholesterol, HDL-c, and fasting glucose were all significantly associated with CIMT. Furthermore, BMI, systolic BP, and total cholesterol were significantly associated with carotid plaque; meanwhile, serum Nrg4 levels showed a borderline significant association with carotid plaque (p = 0.062). After further adjustment for age, sex, current smoking, alcohol consumption, physical activity, HOMA-IR, and body fat, serum Nrg4, systolic BP, total cholesterol, and fasting glucose were significantly associated with CIMT; however, only serum Nrg4 and total cholesterol were significantly associated with carotid plaque in the multivariable regression models.

**Figure 2:**
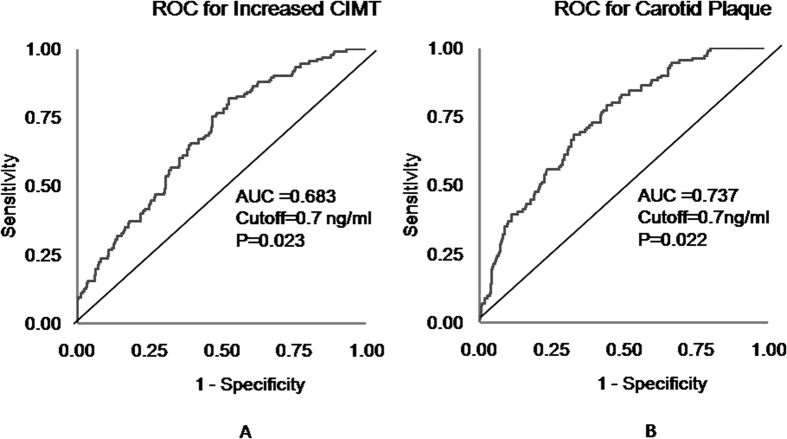
Receiver operating characteristic (ROC) curve for increased carotid intima-media thickness and atherosclerotic plaque according to serum neuregulin 4 levels. (**A**) Receiver operating characteristic (ROC) curve for increased carotid intima-media thickness; (**B**) Receiver operating characteristic (ROC) curve for atherosclerotic plaque; AUC = area under the curve; CIMT = carotid intima-media thickness.

Figure 2 presents results of area under the curve (AUC) calculations for detecting increased CIMT and atherosclerotic plaque according to serum Nrg4 levels. Based on ROC curve analysis, serum Nrg4 levels displayed a significantly high AUC value for detecting increased CIMT (AUC = 0.68, p < 0.05) and atherosclerotic plaque (AUC = 0.74, p < 0.05). The cutoffs of serum Nrg4 levels were 0.71 ng/ml for detecting increased CIMT and 0.69 ng/ml for detecting presence of carotid plaque.

The multivariable-adjusted odds ratios (ORs) for the association of serum Nrg4 levels with increased CIMT and atherosclerotic plaque are shown in Table 4. The risks of increased CIMT and atherosclerotic plaque were reduced by 29% and 31% per 1 SD increase in serum Nrg4 levels (log- transformed), respectively. Importantly, the ORs for increased CIMT and atherosclerotic plaque remained significant [OR (95% CI): 0.72 (0.53–0.98) and 0.69 (0.50–0.96), respectively], even after adjusting for age, sex, current smoking, alcohol consumption, physical activity, BMI, systolic BP, fasting glucose, triglycerides, HDL-c, HOMA-IR, and body fat.

The multivariable-adjusted ORs for increased CIMT and atherosclerotic plaque according to quartiles of serum Nrg4 levels are shown in Table 5. Subjects in the lowest quartile of serum Nrg4 were 3.70 times (p < 0.001) more likely to have increased CIMT and 2.06 times (p < 0.05) more likely to have atherosclerotic plaque than those in the highest quartile. Similarly, subjects in the second and third quartiles also showed significantly elevated risk for increased CIMT and carotid plaque in comparison with those in the fourth quartile (all p < 0.05).

## Discussion

In the present study, we provide for the first time evidence that circulating Nrg4 concentrations were significantly reduced in subjects with subclinical atherosclerotic disease and were inversely associated with increased CIMT and carotid plaque in obese adults. The association of circulating Nrg4 with subclinical atherosclerotic disease persisted after adjustment for metabolic risk factors, body fat, and insulin resistance. These findings indicate that circulating Nrg4 could be a protective factor in the development of CVD.

It has been established that brown adipose tissue is metabolically active in adult humans and correlated with body composition and energy metabolism^18^,^19^,^20^. Activation of brown adipose tissue increases energy expenditure and leads to reduced adiposity, which has provided new insight into the prevention of obesity and metabolic syndrome^14^. Nrg4 was recently identified as a brown-fat-enriched endocrine factor with therapeutic potential for the treatment of obesity-associated disorders, including type 2 diabetes and NAFLD^15^,^21^. Wang *et al*. reported that Nrg4 attenuated hepatic lipogenic signaling and preserved glucose and lipid homeostasis in obesity. However, the potential roles of circulating Nrg4 in contributing to obesity-associated disorders and the associations of circulating Nrg4 with metabolic risk factors remain to be elucidated in population-based studies.

Adipose tissue Nrg4 expression is reduced in several mouse models of obesity and negatively correlates with body fat mass in humans, indicating that Nrg4 insufficiency may be a common feature of obesity^14^,^15^. Obesity is associated with cardiovascular risk factors and increased risk of CVD^5^,^7^. Of note, our data indicated that obese subjects with high levels of circulating Nrg4 had favorable metabolic profiles, including lower levels of BMI, systolic BP, and total cholesterol compared to those with low values. In contrast, Dai and colleague reported that circulating Nrg4 was decreased in NAFLD subjects but not significantly associated with BMI and other metabolic risk factors in a case-control study of 87 NAFLD subjects versus 87 non-NAFLD subjects^22^.

Interestingly, we found that low levels of circulating Nrg4 were independently associated with increased CIMT and atherosclerotic plaque in the present study. Furthermore, our data indicated that each SD increase in circulating Nrg4 levels was associated with a 28% decrease in the risk of increased CIMT and a 30% decrease in the risk of atherosclerotic plaque. Previous research has demonstrated that Nrg4 prevents diet-induced obesity and alleviates obesity-induced insulin resistance in animal models^15^,^23^. In the current study, adjustment for insulin resistance and body fat mass did not attenuate the association of circulating Nrg4 with increased CIMT and atherosclerotic plaque. These findings indicated that low circulating Nrg4 appeared to add to the risk of CVD independently of body fat and HOMA-IR, suggesting that circulating Nrg4 may protect against CVD via mechanisms independent of insulin resistance and obesity. Additionally, our data indicated that circulating Nrg4 might have a cut-off value of 0.7 ng/ml for detecting subclinical atherosclerosis. However, little is currently understood about the role of Nrg4 in the pathophysiology of CVD. It has been proposed that brown adipose tissues is capable of modulating cardiovascular risk factors and atherosclerosis development^18^,^24^,^25^ thus, these data have implications that brown fat-enriched secreted factor Nrg4 may be involved in crosstalk between brown adipose tissue and CVD.

The possible protective role of Nrg4 in subclinical atherosclerosis may involve indirect action, as well as a direct connection between this molecule and the cardiovascular system. In this regard, circulating Nrg4 associated downstream signaling pathways involve the receptor tyrosine kinases ErbB3 and ErbB4, which act as a master transcriptional regulator of cardiomyocyte proliferation and coordinate glucose and lipid homeostasis with thermogenesis^23^,^26^,^27^,^28^. As a novel endocrine factor, Nrg4 acts as an autocrine, paracrine, or endocrine signal by releasing the epidermal growth factor-like domain after photolytic cleavage and activates the receptor kinases ErbB3 and ErbB4 in the cardiovascular system. Therefore, it is possible that Nrg4 may exert a direct effect on the cardiovascular system. However, the effect of Nrg4 in the development of CVD needs to be further studied *in vivo* and *in vitro*.

This community-based, cross-sectional study provided an opportunity to determine the role of circulating Nrg4 in predicting subclinical atherosclerosis. There are several limitations to the current study. First, the study was based on data obtained cross-sectionally with a relatively limited sample size of only obese adults. Therefore, further studies are needed to determine the role of serum Nrg4 in the development of CVD in longitudinal studies. Second, given its cross-sectional design, it is not possible to determine a causal relationship between circulating Nrg4 and the development of increased CIMT and atherosclerosis plaque. In addition, the outcome in our current study was defined as subclinical atherosclerosis rather than clinical cardiovascular events. However, subclinical atherosclerosis measured by ultrasonography is strongly correlated with clinical cardiovascular events and has been used as a surrogate of CVD in an observational study and clinical trial^16^,^17^.

In conclusion, our study provides for the first time clinical evidence revealing that circulating Nrg4 concentrations are inversely associated with increased CIMT and atherosclerosis plaque in obese Chinese adults. These findings suggest that circulating Nrg4 concentrations might play a role in identifying patients at high risk for CVD. However, the role of circulating Nrg4 in the development of CVD needs to be further studied in longitudinal studies.

## Methods

### Study participants

Obese adults aged 40 years or older were screened with a physical examination in the Lianqian community, Xiamen, China from April 2011 to December 2013. The details of the study design and methods have been previously reported^29^. In brief, a total of 485 adult obese subjects (waist circumference ≥ 90 cm for men or 80 cm for women) who received a carotid ultrasound for the measurement of carotid intima-media thickness were included in the analysis. All subjects completed a standard questionnaire including social-demographic status, lifestyle habits (i.e. smoking status, alcohol consumption, and physical activity using the International Physical Activity Questionnaire - Long form), and present and previous health history and medications. Subjects who had cancer, current treatment with systemic corticosteroids, biliary obstructive diseases, acute or chronic virus hepatitis, drug-induced liver diseases, total parenteral nutrition, autoimmune hepatitis, Wilson’s disease, or known hyperthyroidism or hypothyroidism were excluded.

Written informed consent was obtained from each participant. The study protocol was approved by the Institutional Review Board of the First Affiliated Hospital of Xiamen University. The methods were carried out in accordance with the approved guidelines.

### Clinical and biochemical measurements

Anthropometric measurements included height, weight, waist circumference, and blood pressure. Body mass index (BMI, weight in kilograms divided by the square of the height in meters) was used as a measure of adiposity. Waist circumference was measured at the level of the umbilicus. Three measurements were obtained with a nonstretchable tape, and the mean value was used for analysis. Blood pressure (BP) was assessed in triplicate using an electronic sphygmomanometer (OMRON Company). The mean values of the three readings were used for analysis. Body fat mass was determined using the HOLOGIC whole body DXA system (Hologic Inc., Bedford, MA).

75-g oral glucose tolerance tests and blood biochemical measurements were conducted for each subject. All blood samples were obtained after 12 h of fasting. Plasma glucose, triglycerides (TG), total cholesterol (TC), low-density lipoprotein cholesterol (LDL-c), and high-density lipoprotein cholesterol (HDL-c) were measured by enzymatic colorimetric methods with a Hitachi 7450 analyzer (Hitachi, Tokyo, Japan). Fasting plasma glucose concentrations and 2-h glucose concentrations were measured using the glucose oxidase method. Low-density lipoprotein cholesterol (LDL-C) was calculated by Friedewald’s formula. Serum insulin concentrations were measured using electrochemiluminescence immunoassay (Roche Elecsys Insulin Test, Roche Diagnostics, Mannheim, Germany). Insulin resistance status was assessed using the homeostasis model assessment of insulin resistance (HOMA) according to the following formula: fasting serum insulin (μU/mL) × fasting plasma glucose (mmol/L)/22.5.

### Measurement of carotid intima-media thickness

Carotid intima-media thickness (CIMT) measurements were performed by a trained sonographer using a high-resolution B-mode tomographic ultrasound system (Philips-ATL HDI-5000, Philips Medical Systems, Bothell, WA, USA) with a linear 7.5- to 10-MHz transducer. Images were recorded on the far wall of both right and left common carotid arteries at 1.0 cm proximal to the bifurcation^30^. CIMT was measured at the end of diastole as a distance from the leading edge of the first and second lines representing the lumen-intima interface and collagen-contained upper layer of tunic adventitia, respectively. The mean of the maximum CIMT reading of three right and three left far walls for common, bulb, and internal segments was used. Plaque, detected in longitudinal and transverse planes with anterior, lateral, and posterior approaches, was defined as an echogenic focal structure encroaching the vessel lumen with a distinct area 50% greater than the IMT of neighboringsites. Briefly, carotid artery segment, including proximal CCA (0.20 mm proximal to the bulb bifurcation), distal CCA, bulb, ICA, and external carotid artery, were examined bilaterally.

### Serum Nrg4 measurement

Serum Nrg4 concentrations were measured by using enzyme-linked immunosorbentassay (ELISA) kits (Aviscera Biosciences, Santa Clara, CA). The assay has been shown to be highly sensitive to human Nrg4 with a sensitivity of 0.25 ng/ml. The linear range of the standard was 0.5–32.0 ng/ml, and the intra and inter assay variations were both less than 10%.

### Statistical analysis

All statistical analyses were performed with SAS version 9.3 (SAS Institute, Cary, NC). Data are presented as means ± standard deviation (S.D) or means ± standard error (SEM) or median (interquartile range). Data that were not normally distributed were logarithmically transformed before analysis. The subjects were classified into four quartiles according to serum Nrg4 levels or CIMT. The χ^2^-test and logistic regression models were used for comparison of categorical variables between groups. Analyses of covariance were performed using general linear models (GLM) to test differences in study variables between different quartiles of serum Nrg4 levels. Receiver operating characteristic (ROC) curve analysis was applied to evaluate the utility of serum Nrg4 levels in determining subclinical atherosclerosis. Multivariable linear regression models were used to examine the association of serum Nrg4 levels and metabolic risk factors with increased CIMT and atherosclerotic plaque, adjusted for age, sex, smoking, alcohol consumption, physical activity, BMI, systolic BP, glucose, total cholesterol, HDL-c, HOMA-IR, and body fat. Increased CIMT was defined as average CIMT ≥ 0.8 mm^31^,^32^. Multivariable logistic regression models were used to examine the association between serum Nrg4 levels and increased CIMT or atherosclerotic plaque, adjusted for age, sex, smoking, alcohol consumption, physical activity, BMI, systolic BP, glucose, triglycerides, HDL-c, HOMA-IR, and body fat.

## Additional Information

**How to cite this article**: Jiang, J. *et al*. Circulating neuregulin 4 levels are inversely associated with subclinical cardiovascular disease in obese adults. *Sci. Rep.* **6**, 36710; doi: 10.1038/srep36710 (2016).

**Publisher’s note**: Springer Nature remains neutral with regard to jurisdictional claims in published maps and institutional affiliations.

## Supplementary Material

Supplementary Information

## Figures and Tables

**Table 1:** Clinical and biochemical characteristics of obese subjects.

**Variables**	**Overall**	**Male**	**Female**	**P-value**
Sample size	485	131	354	
Age (years)	54.2 ± 7.2	53.8 ± 7.9	54.4 ± 6.9	0.460
BMI (kg/m^2^)	27.2 ± 3.0	27.8 ± 2.5	27.0 ± 3.2	0.017
Waist circumference (cm)	94.2 ± 6.9	97.2 ± 5.7	93.1 ± 7.0	<0.001
Current smokers (n, %)	72 (14.8)	65 (49.6)	7 (2.0)	<0.001
Systolic BP (mmHg)	129.5 ± 16.5	134.4 ± 15.2	127.7 ± 16.6	<0.001
Diastolic BP (mmHg)	77.7 ± 10.1	81.6 ± 9.6	76.3 ± 9.9	<0.001
Triglycerides (mmol/L)	1.6 (1.2–2.2)	1.9 (1.4–2.8)	1.5 (1.1–2.1)	<0.001
Total cholesterol (mmol/L)	5.8 ± 1.0	5.7 ± 0.9	5.8 ± 1.0	0.605
LDL- cholesterol (mmol/L)	3.8 ± 1.0	3.7 ± 0.9	3.8 ± 1.0	0.921
HDL-cholesterol (mmol/L)	1.3 ± 0.3	1.1 ± 0.2	1.4 ± 0.2	<0.001
Fasting glucose (mmol/L)	5.5 ± 0.5	5.6 ± 0.5	5.5 ± 0.5	0.363
2-h glucose (mmol/L)	7.9 ± 2.0	7.5 ± 2.0	8.0 ± 2.0	0.010
HOMA-IR	2.85 (1.95–4.01)	2.84 (2.09–4.04)	2.85 (1.86–4.01)	0.632
Serum Nrg4 (ng/ml)	3.8 (2.7–6.0)	3.2 (2.3–4.9)	3.9 (2.8–6.4)	0.024
Body fat percent (%)	33.9 ± 5.5	27.0 ± 3.4	36.4 ± 3.7	<0.001
CIMT(mm)	0.74 ± 0.16	0.81 ± 0.16	0.71 ± 0.15	<0.001
Atherosclerotic plaque(n, %)	111 (22.9)	39 (29.8)	72 (20.3)	0.012

CIMT = Carotid intima-media thickness; BMI = body mass index; HOMA-IR = homeostasis model assessment of insulin resistance; Nrg4 = Neuregulin 4.

Data are presented as the mean ± SD or median (interquartile range).

**Table 2:** Clinical and biochemical characteristics by quartiles of serum Nrg4 levels.

**Variables**	**Serum Nrg4 level**				**P-value for trend**
	**Q1**	**Q2**	**Q3**	**Q4**	
Sample size	121	121	122	121	
Serum Nrg4 (ng/ml)	2.1 (1.4–2.3)	3.1 (2.9–3.4)	4.5 (4.0–5.2)	8.4 (7.0–11.7)	<0.001
Age (years)	53.7 ± 7.9	54.6 ± 6.9	54.7 ± 6.7	53.9 ± 7.1	0.613
Gender (male n, %)	41 (33.9)	42 (34.7)	24 (19.7)	24 (19.8)	0.005
BMI (kg/m^2^)	27.4 ± 3.3	27.5 ± 2.8	27.4 ± 3.0	26.7 ± 2.9^†^	0.118
Waist circumference (cm)	94.9 ± 7.7	94.0 ± 6.5	94.6 ± 7.0	93.4 ± 6.4	0.321
Current smokers (n, %)	21 (17.4)	21 (17.4)	16 (13.1)	14 (11.6)	0.471
Physical activity (met/h. week)	23.1 (9.6–46.2)	24.0 (11.0–57.4)	23.1 (10.0–41.2)	23.1 (5.8–49.5)	0.568
Systolic BP (mmHg)	131.7 ± 17.0	131.1 ± 13.9	128.7 ± 17.3	126.7 ± 17.2^†^	0.066
Diastolic BP (mmHg)	78.8 ± 9.7	78.9 ± 8.9	76.7 ± 10.4	76.5 ± 11.2	0.111
Triglycerides (mmol/L)	1.6 (1.2–2.2)	1.6 (1.2–2.3)	1.6 (1.2–2.3)	1.5 (1.1–2.1)	0.492
Total cholesterol (mmol/L)	5.8 ± 1.0	5.8 ± 1.0	5.9 ± 1.0	5.5 ± 1.0^†^	0.072
LDL- cholesterol (mmol/L)	3.8 ± 1.0	3.7 ± 1.0	3.8 ± 1.0	3.6 ± 0.9	0.327
HDL-cholesterol (mmol/L)	1.3 ± 0.2	1.3 ± 0.3	1.3 ± 0.3	1.3 ± 0.3	0.462
Fasting glucose (mmol/L)	5.6 ± 0.5	5.6 ± 0.6	5.5 ± 0.5	5.5 ± 0.5	0.203
2-h glucose (mmol/L)	7.6 ± 1.9	7.7 ± 1.9	8.2 ± 2.1^‡^	7.9 ± 2.0	0.097
HOMA-IR	2.98 (1.93–4.08)	2.88 (2.10–4.01)	2.80 (1.80–3.89)	2.70 (1.94–4.04)	0.958
Body fat percent (%)	33.6 ± 5.9	33.1 ± 5.9	34.9 ± 4.8^†^	33.9 ± 5.5	0.070
CIMT(mm)	0.78 ± 0.17	0.77 ± 0.14	0.73 ± 0.15^‡^	0.66 ± 0.12^‡^	<0.001
Atherosclerotic plaque(n, %)	32 (26.4)	30 (24.8)	30 (24.6)	19 (15.7)^†^	0.040

CIMT = Carotid intima-media thickness; BMI = body mass index; HOMA-IR = homeostasis model assessment of insulin resistance; Nrg4 = Neuregulin 4.

Data are presented as the mean ± SD or median (interquartile range).

^†^P < 0.05 compared with Q1.

^‡^P < 0.01 compared with Q1.

**Table 3:** Standardized regression coefficients of serum Nrg4 levels and metabolic risk factors for predicting carotid intima-media thickness and atherosclerotic plaque.

**Independent variables**	**Unadjusted**			**Adjusted**^**§**^		
	**β**	**SE**	**P-value**	**β**	**SE**	**P-value**
CIMT						
BMI	0.0001	0.007	0.984	−0.002	0.009	0.867
Systolic BP	0.031	0.007	<0.001	0.023	0.007	0.001
Total cholesterol	0.031	0.007	<0.001	0.025	0.007	<0.001
HDL-cholesterol	−0.024	0.007	<0.001	−0.014	0.008	0.069
Fasting glucose	0.026	0.007	<0.001	0.022	0.008	0.004
Serum Nrg4	−0.024	0.006	<0.001	−0.022	0.006	<0.001
Model R^2^ (%)	0.194			0.267		
Atherosclerotic plaque						
BMI	−0.045	0.020	0.030	−0.046	0.027	0.088
Systolic BP	0.055	0.021	0.008	0.021	0.021	0.315
Total cholesterol	0.068	0.021	0.001	0.054	0.021	0.008
HDL-cholesterol	−0.039	0.021	0.062	−0.037	0.022	0.103
Fasting glucose	0.008	0.021	0.440	−0.026	0.022	0.238
Serum Nrg4	−0.036	0.019	0.062	−0.039	0.019	0.037
Model R^2^ (%)	0.061			0.156		

CIMT = carotid intima-media thickness; BMI = body mass index; HOMA-IR = homeostasis model assessment of insulin resistance; Nrg4 = Neuregulin 4; β = standardized regression coefficient; SE = standard error.

^§^Adjusted for age, sex, smoking, physical activity, HOMA-IR, and body fat.

**Table 4:** Odds ratios for increased carotid intima-media thickness and atherosclerotic plaque according to serum Nrg4 levels.

	**Increased CIMT**		**Atherosclerotic plaque**	
	**OR (95% CI)**	**P-value**	**OR (95% CI)**	**P-value**
Model 1	0.71 (0.53–0.95)	0.022	0.69 (0.50–0.95)	0.022
Model 2	0.72 (0.53–0.98)	0.039	0.70 (0.50–0.97)	0.034
Model 3	0.72 (0.53–0.98)	0.037	0.69 (0.50–0.96)	0.028

OR = odds ratio; CI = confidence interval; BMI = body mass index; HOMA-IR = homeostasis model assessment of insulin resistance; CIMT = carotid intima-media thickness; Nrg4 = Neuregulin 4.

Model 1: adjusted for age, sex, smoking, and physical activity.

Model 2: adjusted for model 1 + adjusted for metabolic components, including BMI, SBP, glucose, triglycerides, and HDL-c.

Model 3: adjusted for model 2 + adjusted for HOMA-IR and body fat.

**Table 5:** Odds ratios for increased carotid intima-media thickness and atherosclerotic plaque according to quartiles of serum Nrg4 levels, adjusted for covariates^§^ in logistic regression models.

	**Increased CIMT**		**Atherosclerotic plaque**	
	**OR (95% CI)**	**Multivariate-adjusted P-value**	**OR (95% CI)**	**Multivariate-adjusted P-value**
Q1 vs. Q4	3.70 (1.85–7.41)	—	2.06 (1.02–4.16)	—
Q2 vs. Q 4	2.72 (1.36–5.43)	—	1.78 (0.88–3.58)	—
Q3 vs. Q 4	2.32 (1.14–4.72)	—	1.84 (0.91–3.72)	—
(Q1 + Q2 + Q3) vs. Q 4	2.87 (1.57–5.25)	<0.001	1.89 (1.04–3.41)	0.036

OR = odds ratio; CI = confidence interval; CIMT = carotid intima-media thickness; Nrg4 = Neuregulin 4; Q = quartile.

^§^Adjusted for age, sex, smoking, physical activity, BMI, systolic BP, glucose, total cholesterol, HDL-c, HOMA-IR, and body fat.
