# Supplementary material for: Circulating neuregulin 4 levels are inversely associated with subclinical cardiovascular disease in obese adults
Source: Sci Rep. 2016 Nov 7;6:36710. doi: 10.1038/srep36710 (PMC5098181; doi:10.1038/srep36710)

**Circulating neuregulin 4 levels are inversely associated with subclinical cardiovascular disease in obese adults**

Jie Jiang^1^*, Mingzhu Lin^2^*, Yanfang Xu^3^, Jin Shao^2^, Xuejun Li^2^, Huijie Zhang^2 4^, Shuyu Yang^2^

Supplemental Figure 1 Serum neuregulin 4 levels by quartiles of carotid intima-media thickness

* P< 0.05 compared with Q1; ** P< 0.01 compared with Q1


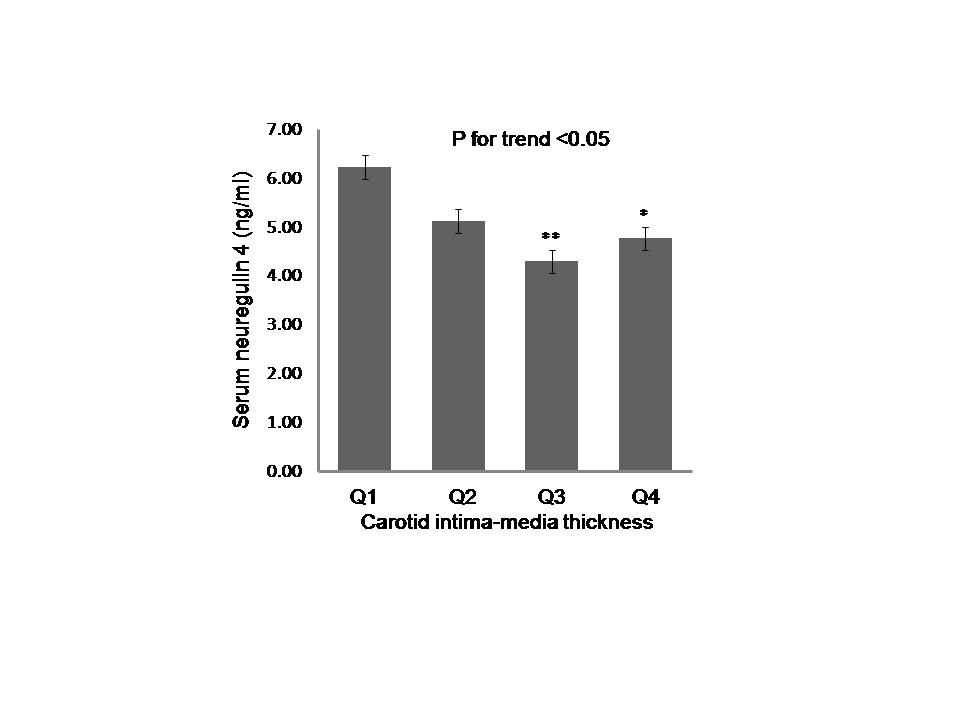


Supplemental Figure 2 Serum neuregulin 4 levels by presence of atherosclerotic plaque


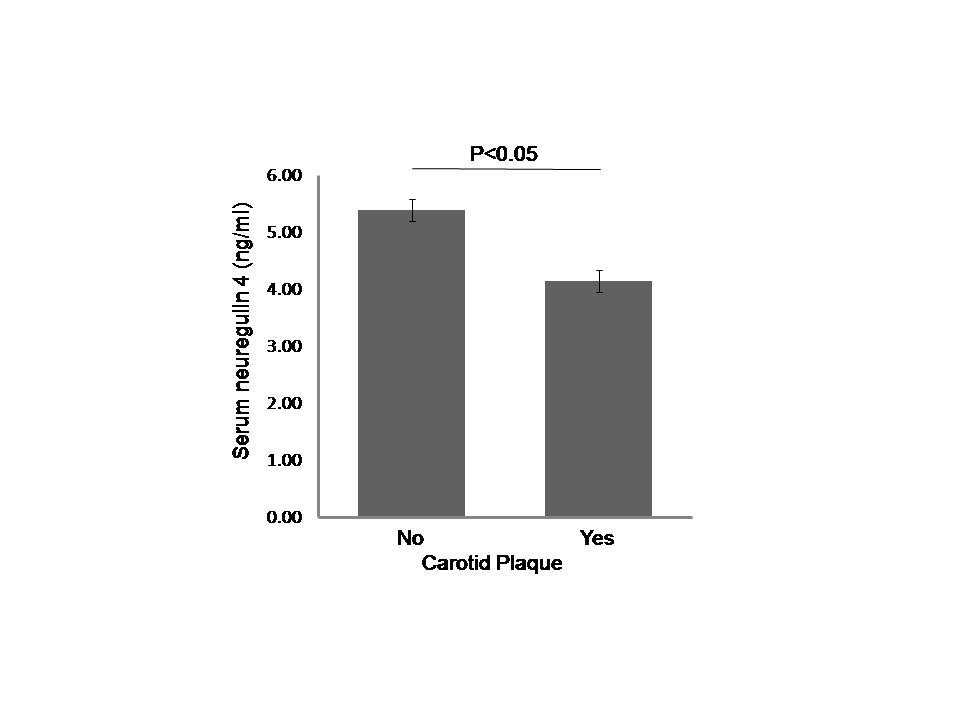

Supplement: Supplementary Information [file srep36710-s1.doc]
